# Genomic microdeletions associated with epilepsy: Not a contraindication to resective surgery

**DOI:** 10.1111/j.1528-1167.2011.03087.x

**Published:** 2011-08

**Authors:** Claudia B Catarino, Dalia Kasperavičiūtė, Maria Thom, Gianpiero L Cavalleri, Lillian Martinian, Erin L Heinzen, Thomas Dorn, Thomas Grunwald, Elijah Chaila, Chantal Depondt, Günter Krämer, Norman Delanty, David B Goldstein, Sanjay M Sisodiya

**Affiliations:** * Department of Clinical and Experimental Epilepsy, UCL Institute of Neurology and National Hospital for Neurology and NeurosurgeryQueen Square, London, United Kingdom; † National Society for Epilepsy, Chalfont St PeterBucks, United Kingdom; ‡ Division of Neuropathology, Department of Clinical and Experimental Epilepsy, UCL Institute of Neurology and National Hospital for Neurology and NeurosurgeryQueen Square, London, United Kingdom; § Molecular and Cellular Therapeutics, The Royal College of Surgeons in IrelandSt. Stephens Green, Dublin, Ireland; ¶ Institute for Genome Sciences & Policy, Center for Human Genome Variation, Duke UniversityDurham, North Carolina, U.S.A.; ** Swiss Epilepsy CentreBleulerstrasse 60, Zurich, Switzerland; †† Department of NeurologyHôpital Erasme, Université Libre de Bruxelles, Brussels, Belgium

**Keywords:** Epilepsy surgery, Hippocampal sclerosis, Temporal lobectomy, Deletions

## Abstract

**Purpose:**

Several recent reports of genomic microdeletions in epilepsy will generate further research; discovery of more microdeletions and other important classes of variants may follow. Detection of such genetic abnormalities in patients being evaluated for surgical treatment might raise concern that a genetic defect, possibly widely expressed in the brain, will affect surgical outcome.

**Methods:**

A reevaluation was undertaken of clinical presurgical data, histopathology of surgical specimen, and postsurgical outcome in patients with mesial temporal lobe epilepsy (MTLE) who have had surgical treatment for their drug-resistant seizures, and who have been found to have particular genomic microdeletions.

**Key Findings:**

Three thousand eight hundred twelve patients with epilepsy were genotyped and had a genome-wide screen to identify copy number variation. Ten patients with MTLE, who had resective epilepsy surgery, were found to have 16p13.11 microdeletions or other microdeletions >1 Mb. On histopathology, eight had classical hippocampal sclerosis (HS), one had nonspecific findings, and one had a hamartoma. Median postsurgical follow-up time was 48 months (range 10–156 months). All patients with HS were seizure-free after surgery, International League Against Epilepsy (ILAE) outcome class 1, at last follow-up; the patient with nonspecific pathology had recurrence of infrequent seizures after 7 years of seizure freedom. The patient with a hamartoma never became seizure-free.

**Significance:**

Large microdeletions can be found in patients with “typical” MTLE. In this small series, patients with MTLE who meet criteria for resective surgery and harbor large microdeletions, at least those we have detected, can have a good postsurgical outcome. Our findings add to the spectrum of causal heterogeneity of MTLE + HS.

Recurrent genomic microdeletions have recently been identified in “common” sporadic epilepsies, those not obviously part of a genetic syndrome. 15q13.3, 15q11.2, and 16p13.3 microdeletions together underlie ∼3% of idiopathic generalized epilepsies (IGEs) (Helbig et al., 2009; de Kovel et al., 2010; Mefford et al., 2010). We identified 16p13.11 and other large microdeletions in ∼0.8% of patients with epilepsy (Heinzen et al., 2010). As more microdeletions are reported and technologies for the detection of these and other putatively causal variants become more accessible, interest in searching for such variants will increase.

A number of patients in our initial cohort had mesial temporal lobe epilepsy (MTLE) and had resective epilepsy surgery, and some were found to harbor particular microdeletions. The detection of a microdeletion might raise concern about its potential influence on outcome measures following epilepsy surgery, with regard to seizure control or other domains. The presence of a microdeletion could represent widespread brain involvement, similar to cognitive impairment or secondary generalized tonic–clonic seizures, both of which reduce chances of good outcome across various domains after epilepsy surgery (Malmgren et al., 2008; Spencer & Huh, 2008). On the other hand, such microdeletions might not affect outcome, for example because of spatial variability in gene expression (Hardy et al., 2009). We evaluated systematically the effect of large microdeletions on outcome after surgery in patients with MTLE.

## Methods

This work was approved by the relevant local research ethics committees. All patients provided written informed consent.

Microdeletions were identified as described previously (Heinzen et al., 2010). Only microdeletions >1 Mb, or 16p13.11 microdeletions, were considered (Heinzen et al., 2010). We reevaluated clinical history and presurgical investigations [magnetic resonance imaging (MRI) brain scan, video–electroencephalography (EEG) telemetry, neuropsychometry, neuropsychiatric assessment]. Histopathology of the surgical specimen was reviewed. Postsurgical outcome was evaluated in terms of seizure control at 1 year and at last follow-up, using the International League Against Epilepsy (ILAE) outcome classification (Wieser et al., 2001), antiepileptic drug changes, psychiatric outcome, neuropsychometry, and employment outcome.

## Results

Three thousand eight hundred twelve patients with epilepsy (>90% with partial epilepsies) were genotyped and had a genome-wide screen to identify copy number variation. Ten patients with MTLE who had undergone therapeutic resection had large microdeletions. Follow-up duration after surgery ranged from 10–156 months. Three patients had 16p13.11 microdeletion and two had 15q11.2 microdeletion; the full range of microdeletions is listed in Table 1.

**Table 1:** List of heterozygous 16p13.11 microdeletions or other microdeletions >1 Mb in MTLE patients who had resective surgery

Case ID	Cytoband	Breakpoints	Size (Mb)	Gene list

1	16p13.11	chr16:15387380–16225138	0.8	*MPV17L*, *C16orf45*, *NDE1*, *MYH11*, *C16orf63*, *ABCC1*, *ABCC6*
2	16p13.11	chr16:15387380–16225138	0.8	*MPV17L*, *C16orf45*, *NDE1*, *MYH11*, *C16orf63*, *ABCC1*, *ABCC6*
3	7q31.32–31.33	chr7:123252578–126117199	2.9	*HYAL4*, *SPAM1*, *LOC136157*, *GPR37*, *POT1*, *GRM8*
4	17p12	chr17:14040467–15411904	1.4	*COX10*, *CDRT15*, *HS3ST3B1*, *PMP22*, *TEKT3*, *CDRT4*, *FAM18B2*
5	4q32.3	chr4:167446375–168643447	1.2	*SPOCK3*
6	17q12	chr17:31922987–33333394	1.4	*ZNHIT3*, *MYO19*, *PIGW*, *GGNBP2*, *DHRS11*, *MRM1*, *LHX1*, *AATF*, *ACACA*, *C17orf78*, *TADA2L*, *DUSP14*, *AP1GBP1*, *DDX52*, *HNF1B*, *LOC284100*
7	15q11.2	chr15:18285782–20868229	1.3	*OR4N4*, *NIPA2*, *NIPA1*, *TUBGCP5*, *CYFIP1*, *HERC2P2*, *A26B1 (POTEB)*, *OR4M2*, *AC131280.9*, *AC126603.9*, *AC116165.7*, *AC026495.13*, *AC025884.28*, *AC138701.3*, *AC127381.4*, *AC126335.16*, *AC091565.10*, *AC134980.3*, *AC138649.2*
8	15q11.2	chr15:18822307–19852603	1.0	*AC025884.28*, *AC026495.13*, *OR4N4*, *OR4M2*, *AC131280.9*, *AC134980.3*, *AC126335.16*, *A26B1*
9	4q35.2	chr4:189052964–190737252	1.97	*AC093909.2*, *AC020698.4*, *TRIML2*, *TRIML1*, *ZFP42*
10	16p13.11	chr16:15387380–16198600	0.8	*MPV17L*, *C16orf45*, *NDE1*, *MYH11*, *C16orf63*, *ABCC1*, *ABCC6*

Demographic and clinical data are summarized in (Table S1), including details of the type of surgery and outcome of surgery across several domains, including seizure control. The histopathologic results from analysis of the surgical specimen are listed in Table 2.

**Table 2:** Main findings of neuropathology analysis of temporal lobectomy specimen

Case ID	Main pathologic findings in temporal neocortex	Main pathologic findings in hippocampus	Summary of main pathologic findings

1	Small glioneuronal hamartoma in middle temporal gyrus white matter	Only CA1 available for analysis; neuronal loss not seen	No hippocampal sclerosis but specimen incomplete. Hamartoma
2	Focal neuronal loss and gliosis in superficial cortex in pole (TLS) (Thom et al., 2009)	Neuronal loss and gliosis particularly in CA1 and CA4. Mild GCD	Classical hippocampal sclerosis
3	Cortex normal	Neuronal loss and gliosis particularly in CA1 and CA4. Moderate GCD	Classical hippocampal sclerosis
4	Gliosis only. No dysplasia	Incomplete representation of subfields. CA1 neuronal loss and gliosis. GCD	Classical hippocampal sclerosis
5	Patchy cortical and white matter gliosis	Neuronal loss and gliosis particularly in CA1 and CA4. Moderate GCD	Classical hippocampal sclerosis
6	Cortex normal	Neuronal loss in CA4 and CA1 and gliosis	Classical hippocampal Sclerosis
7	Numerous corpora amylacea in white matter	Incomplete representation of subfields. CA1 and CA4 neuronal loss and gliosis. Mild GCD and some depletion of GC	Classical hippocampal sclerosis
8	N/A*a*	Neuronal loss and gliosis particularly in CA1 and CA4	Classical hippocampal sclerosis.
9	N/A*a*	Moderate to marked astrogliosis	Classical hippocampal sclerosis
10	Patchy laminar reactive astrogliosis	Amygdala included, but hippocampal structures not present in specimen*b*	Nonspecific findings

a These patients had selective amygdalohippocampectomy.

b This patient had neocorticectomy and amygdalectomy.

GC(D), Granule cell (dispersion); N/A, not applicable; TLS, temporal lobe sclerosis (Thom et al., 2009).

Eight patients had histologically proven classical hippocampal sclerosis (HS) (Table 2). All were rendered seizure-free after surgery. All displayed clinical features “typical” of MTLE + HS (Wieser, 2004) (Table S1). In all patients except one, antiepileptic drugs (AEDs) were reduced in number and/or daily dose during long-term follow-up. Two patients were off AEDs; they had remained seizure-free at last follow-up.

Another patient, with MRI-negative temporal lobe epilepsy, had a right neocorticectomy and amygdalectomy, with nonspecific findings at histopathology, and after 7 years of seizure freedom, began again to have infrequent partial seizures. One patient with a hamartoma had a right anterior temporal lobectomy, but was never rendered seizure-free. There were no unexpected findings in other domains during postsurgical follow-up.

## Discussion

In patients with drug-resistant MTLE + HS, surgery is more effective in stopping disabling seizures than medical treatment alone (Wiebe et al., 2001). Recent studies also suggest that such surgery can benefit longevity (Choi et al., 2008) and quality of life (Zupanc et al., 2010). Around one-third of patients who undergo surgery fail to become seizure-free, the causes for which are uncertain; the proportion not seizure-free increases at longer-term follow-up (Wieser, 2004). Although several predictors of outcome are known or proposed at a group level, including presence of HS, absence of generalized tonic–clonic seizures, larger extent of mesiotemporal resection, and unilateral interictal epileptiform activity (Wyler et al., 1995; Radhakrishnan et al., 1998; Engel et al., 2003; Tonini et al., 2004; Janszky et al., 2005; Spencer et al., 2005; Spencer & Huh, 2008), there are no universally accepted outcome predictors for MTLE + HS at an individual level. Such predictors would assist in estimating risk/benefit ratio for each patient more accurately.

Genetic factors might be postulated to contribute to outcome, and indeed to causation of MTLE + HS. Familial MTLE exists (Berkovic et al., 1994, 1996; Crompton et al., 2010). Some argue that familial MTLE with HS/hippocampal atrophy is clinically indistinguishable from sporadic MTLE + HS (Kobayashi et al., 2003a; Gambardella et al., 2009). No genetic cause of familial MTLE is known. In addition, no genetic determinant for susceptibility to sporadic MTLE + HS, or for predicting outcome after epilepsy surgery, has been confirmed (Kanemoto et al., 2000; Stögmann et al., 2002; Cavalleri et al., 2005, 2007; Kasperaviciute et al., 2010).

Microdeletions are becoming recognized as an important potential genetic cause of, or predisposition to, a wide range of epilepsies. Although some microdeletions may be chance findings, large microdeletions are likely to be associated with disease (Scheffer & Berkovic, 2010), and we showed changes in gene expression with 16p13.11 microdeletions, and noted that many known epilepsy genes, for example, *KCNA1*, *GABRA1*, and *GABRG2*, can be involved in microdeletions (Heinzen et al., 2010). It is important to note that many of these microdeletions seem to act as risk factors rather than as the sole underlying cause, and that some microdeletions are found in people without epilepsy or a family history of epilepsy, although at much reduced frequency (Sisodiya & Mefford, 2011). Although more work is required to characterize microdeletions and their pathogenic mechanisms, significant microdeletions are likely to be found in many other patients, including those being considered for epilepsy surgery. Because these microdeletions might affect outcome after surgical treatment, it is important to systematically evaluate their influence.

The seizure-free rate in our patients with putatively pathogenic microdeletions seems as good as reported for cohorts in the literature (McIntosh et al., 2001; Spencer & Huh, 2008; Dunlea et al., 2010). Although this observation must be tempered by the small size of our microdeletion cohort and requires further confirmation, we show that having a large microdeletion does not preclude seizure-free outcome after surgery for MTLE. Although our findings may relate to specific microdeletions, our seizure-free patients had seven different microdeletions, and had a good postsurgical outcome in other domains as well, not just seizure control (Table S1). The psychiatric outcome varied, with presurgical psychiatric comorbidity common in the cases with postsurgical psychiatric issues, as reported previously in the literature (Kanner et al., 2009).

There are other examples in the literature of epilepsies with a genetic basis and a good outcome after resective surgery for drug-resistant seizures. In families with *SCN1B*-positive genetic epilepsy with febrile seizures plus (GEFS+), an excellent outcome has been reported after anterior temporal lobectomy for affected individuals with drug-resistant MTLE + HS (Scheffer et al., 2007). A small series of patients with tuberous sclerosis complex, with confirmed mutations in the *TSC1* (n = 2) and *TSC2* (n = 2) genes, had discrete epileptogenic brain lesions that were resected, and were seizure-free on AEDs at last follow-up after surgery (Hirfanoglu & Gupta, 2010). Postsurgical seizure outcome in patients with familial MTLE with HS/hippocampal atrophy, for which no known genetic cause is actually yet known, does not differ from that in sporadic MTLE with HS/hippocampal atrophy (Kobayashi et al., 2003b).

Our findings suggest that large microdeletions do not necessarily preclude a good prognosis following epilepsy surgery, if surgery is a reasonable option based on concordance of other data during presurgical evaluation. Further studies will be important to firmly establish the mechanisms of MTLE associated with large microdeletions. As more putatively causal genetic variants of all classes are uncovered, it will also become important to address their impact on clinical management.

## Supporting Information

**Table S1.** Clinical data, including preoperative investigations, type of surgery, and post-surgical outcome.
