# Supplementary material for: Genomic microdeletions associated with epilepsy: Not a contraindication to resective surgery
Source: Epilepsia. 2011 Aug;52(8):1388–92. doi: 10.1111/j.1528-1167.2011.03087.x (PMC3399084; doi:10.1111/j.1528-1167.2011.03087.x)
Supplement: Supplementary file 1 [file epi0052-1388-SD1.doc]

| **Case ID** | **Gender** | **Decade of onset of habitual seizures** | **Febrile seizures** | **Seizure types** | **Video-EEG interictal findings** | **Video-EEG ictal findings** | **MRI brain scan** | **Duration of epilepsy to surgery (y)** | **Surgery type** | **Duration of**  **postop follow-up (mo)** | **Surgical outcome: ILAE outcome class / current number of AEDs/ preop number of AEDs** | **Cognitive / psychiatric / employment post-surgical outcome** |
| --- | --- | --- | --- | --- | --- | --- | --- | --- | --- | --- | --- | --- |
| **1** | M | third | NO | SPS, CPS, SGTCS | R temporal IED, L frontal slow | R temporal onset | Hippocampal asymmetry | 8 | R ATLx | 76 | ILAE class 5/ on 1AED/ preop 1AED | good memory outcome / no psychiatric issues / works |
| **2** | F | first | YES | CPS, SGTCS | L ant-mid temporal IED | L temporal onset | L HS | 34 | L ATLx | 36 | ILAE class 1/ on 2AED/ preop 3AED | psychometry no significant changes from preop / reactive depression / works |
| **3** | M | first | NO | CPS | L ant-mid temporal IED | no seizures recorded | L HS | 41 | L ATLx | 36 | ILAE class 1/ on 1AED/ preop 2AED | psychometry no significant changes from preop / no psychiatric issues / works |
| **4** | M | first | YES | SPS, CPS, SGTCS | L ant-mid temporal IED | not lateralised | L HS | 30 | L ATLx | 96 | ILAE class 1/ on 3AED/ preop 3AED | some verbal memory problems but non-verbal memory improved / reactive depression resolved / works full-time |
| **5** | M | fourth | YES | CPS, rare SGTCS | L temporal IED and slow | L temporal onset | L HS | 3 | L ATLx | 60 | ILAE class 1/ on 1AED/ preop 2AED | good memory outcome / no psychiatric issues / works full-time |
| **6** | M | first | NO | SPS, CPS | R temporal IED | R temporal onset | R HS | 18 | R ATLx | 15 | ILAE class 1/ on 1AED/ preop 2AED | improved attention span and verbal memory, slight decline of visual memory / no psychiatric issues / works |
| **7** | F | first | NO | SPS, SGTCS | L temporal IED and slow | L temporal onset | L HS | 24 | L ATLx | 10 | ILAE class 1/ on 1AED/ preop 2AED | cognitive outcome good (verbal recall and visual memory improved / no psychiatric issues |
| **8** | F | first | YES | SPS, CPS, SGTCS | L temporal IED, bilateral unspecific slow | L temporal onset | L HS | 32 | L selective AHx | 36 | ILAE class 1/ on 1AED / preop 2 AEDs | awaits postop neuropsychological assessment / no postop change in psychiatric or employment status |
| **9** | F | first | NO | SPS, CPS | N/A | R temporal onset (depth recordings) | R hippocampal atrophy | 40 | R selective AHx | 72 | ILAE class 1/ off AEDs | N/A |
| **10** | M | third | Not known | CPS, SGTCS | R temporal IED and slow | R temporal onset (sphenoidal electrodes) | Normal | 8 | R neocorticectomy and Ax | 156 | ILAE class 1 for 7yrs, then class 3/ on 2 AEDs/ preop 3 AEDs | cognitive outcome good / no psychiatric issues / no postop change in employment status |

Table S1. Clinical data, including preoperative investigations, type of surgery, and post-surgical outcome. Abbreviations: AEDs=antiepileptic drugs; Ax = amygdalectomy; AHx = amygdalo-hippocampectomy; ant-mid = antero-mid; ATLx = anterior temporal lobectomy with amygdalo-hippocampectomy; CPS = complex partial seizures; DZ =dizygotic; F = female; HS = hippocampal sclerosis; IED = interictal epileptiform discharges; L = left; LD = learning difficulties; M = male; mo = months; N/A = not applicable or not available; postop = postoperative; R = right; SGTCS = secondary generalised tonic-clonic seizures; SPS = simple partial seizures; y=years.
